# The impact of COVID‐19 severity on adult survivors: Is there a relationship between vascular reactivity and cardiorespiratory fitness?

**DOI:** 10.14814/phy2.70216

**Published:** 2025-03-02

**Authors:** Guilherme Dionir Back, Murilo Rezende Oliveira, Guilherme Peixoto Tinoco Arêas, Patricia Faria Camargo, Cássia da Luz Goulart, Claudio Ricardo de Oliveira, José Carlos Bonjorno Junior, Flávia Rossi Caruso Bonjourno, Ross Arena, Audrey Borghi‐Silva

**Affiliations:** ^1^ Cardiopulmonary Physiotherapy Laboratory, Physiotherapy Department Federal University of São Carlos, UFSCar São Carlos Brazil; ^2^ Exercise Physiology Laboratory, College of Physical Education and Physiotherapy (FEFF) Federal University of Amazonas Manaus Brazil; ^3^ College of Physical Education University of Brasília Brasília Brazil; ^4^ Department of Physical Therapy, College of Applied Health Sciences University of Illinois Chicago Chicago Illinois USA

**Keywords:** cardiopulmonary testing, COVID‐19, endothelial function, survivors

## Abstract

The impact of COVID‐19 on cardiorespiratory fitness (CRF) is negative, increasing the likelihood of exertional symptoms such as fatigue and shortness of breath, and adversely affecting vascular function, impairing cardiovascular health. This study investigated endothelial function and its relationship with CRF in patients who have recovered COVID‐19. Patients were evaluated 1 month after infection, including clinical assessment, pulmonary function, endothelial function (measured by flow‐mediated dilation), and cardiopulmonary exercise testing. COVID‐19 survivors exhibited reduced exercise capacity, with poor values of peak V̇O_2peak_ and FMD (%) according to disease severity. However, endothelial function was worse in COVID‐19 patients, regardless of severity, compared to the control group. Significant associations were observed between poorer FMD (%) and peak V̇O_2_, workload, circulatory power, and V̇O_2peak_/WR. Endothelial function was significantly associated with CRF in COVID‐19 patients according to disease severity. Strategies to improve CRF and reduce the negative impacts of endothelial function damage should be further investigated.

## 1 INTRODUCTION

The coronavirus disease 2019 (COVID‐19) pandemic precipitated a series of unprecedented challenges for global health and society as a whole (Borghi‐Silva et al., 2022). As the pandemic progressed, it became increasingly evident that severe acute respiratory syndrome coronavirus 2 (SARS‐CoV‐2) was not just a respiratory virus but a virus that also affected multiple organ systems, including the cardiovascular system. (Silva et al., 2021). Endothelial dysfunction, an important marker of cardiovascular diseases, emerged as a common feature in COVID‐19 patients, and the long‐term impacts of this dysfunction in those recovering from viral infection became a growing concern (Oliveira et al., 2021).

The relationship between endothelial dysfunction and cardiorespiratory fitness (CRF) has been a subject of investigation, with studies demonstrating that the integrity of the vascular endothelium plays a fundamental role in the body's ability to respond to physical stress and maintain the balance between oxygen supply and demand during exercise (Dobrosielski et al., 2016; Sen et al., 2023). However, investigation into the endothelial dysfunction‐CRF relationship in COVID‐19 survivors is currently limited. Among a range of clinical manifestations related to SARS‐CoV‐2 infection, an impaired exercise response in COVID‐19 survivors, assessed through cardiopulmonary exercise testing (CPET), which is the gold standard for assessing CRF, has been reported (Rinaldo et al., 2021; Skjørten et al., 2021). In this context, the European Respiratory Society/American Thoracic Society (ERS/ATS) task force recommended CPET for COVID‐19 follow‐up as a well‐established diagnostic tool to assess symptom etiology and underlying mechanisms that limit exercise in cardiovascular and pulmonary diseases (Bai et al., 2020).

Given the fundamental role of vascular endothelial integrity in maintaining the homeostasis of the cardiovascular and respiratory systems (Vane et al., 1990), this study aimed to evaluate endothelial function and investigate its relationship to CRF in patients who have recovered from mild to severe COVID‐19. As such, the current investigation seeks to fill a significant gap in the literature by providing further insights into the relationship between endothelial dysfunction and CRF following COVID‐19 and its impact on the ability to perform physically demanding activities.

## 2 MATERIALS AND METHODS

### 2.1 Design and ethical approval

This cross‐sectional study followed recommendations of the STROBE declaration. Patients gave written informed consent to participate to the research protocol, approved by the Institutional Medical Ethics Committee (protocol number: 32408720.5.0000.5504), and adhered to Resolution 466/2012 of the National Health Council and followed the ethical guidelines of the Declaration of Helsinki (1975). All data collected by the researchers were from real situations experienced by the patients, and no data were generated by artificial intelligence in the present study.

### 2.2 Subjects

Subjects of both sexes were included if they were: (1) positive for COVID‐19 based on nasal swab real‐time reverse transcriptase‐polymerase chain reaction; and (2) aged between 18 and 60 years. The severity of the acute illness was defined by the provisional clinical guidance of the World Health Organization (WHO) (Diaz et al., 2021). Mild cases were classified as: (1) flu syndrome with mild symptoms (no dyspnea or signs of severity, without evidence of viral pneumonia or hypoxia); (2) absence of decompensated comorbidity without the need for hospitalization; and (3) home isolation for at least 10 days. Moderate cases were defined as: (1) moderate symptoms with clinical signs of pneumonia (fever, cough, dyspnea, rapid breathing that required stabilization and admission to the ward, non‐invasive ventilatory support); (2) no signs of severe pneumonia, including SpO_2_ ≥90% on room air; (3) involvement of ≤50% of the lung parenchyma on computed tomography; (4) hospitalization ≤10 days; and (5) respiratory and motor physiotherapy at least once a day. Severe cases were defined as the requirement for Intensive Care Unit (ICU) admission with or without mechanical ventilation (MV) and non‐invasive ventilation, pulmonary involvement of less than 50% of the lung parenchyma, and pneumonia‐like signs such as SpO_2_ <88% in room air.

Exclusion criteria were: (1) absence of informed consent; (2) over 60 years of age; (3) acute respiratory exacerbation within 4 weeks before enrollment; (4) use of home oxygen; (5) use of illicit drugs or alcohol; (6) pregnancy; and (7) dementia.

### 2.3 Experimental procedures

Subjects were selected and recruited by members of the research team who visited the hospital weekly. Hospitalized patients were followed during hospitalization and after hospital discharge through telephone calls on the 20th day. Survivors in home quarantine were allocated from a list of test results provided by the hospitals. Patients who survived COVID‐19 underwent a clinical evaluation divided into two consultations: (1) clinical assessment, pulmonary function, and flow‐mediated dilation; (2) CPET. All evaluations were conducted at 48 h intervals between each laboratory visit. Research team members were properly equipped with personal protective equipment, including a waterproof apron, goggles, latex gloves, N95 mask, disposable mask, disposable cap, and face shield. In addition, the entire physical space was adapted according to current recommendations (Diaz et al., 2021).

### 2.4 Clinical evaluation

The patients who recovered from COVID‐19 were assessed 1 month after the acute phase of infection. All volunteers were contacted by phone 48 h prior to the assessment. During the screening, subjects reported any COVID‐19 symptoms experienced in the last 10 days. The following evaluations were conducted: clinical evaluation, pulmonary function, flow‐mediated dilatation, and CPET.

### 2.5 Evaluations and measurements

Clinical data including age, sex, weight, height, race, information on past medical history, comorbidities, persistent symptoms, smoking, and medication use were collected to characterize the sample.

### 2.6 Pulmonary function

The variables analyzed were forced expiratory volume in 1 s (FEV_1_), functional residual capacity (FRC), and FVC/FEV_1_, by the MasterScreen™ Body Plethysmograph (Mijnhardt/Jäguer, Germany). Spirometry was performed according to the recommendations of the American Thoracic Society/European Respiratory Society (Miller et al., 2005), and the results were compared to previously described reference values (Pereira et al., 2007).

### 2.7 Flow‐mediated dilation (FMD)

For the assessment of FMD, patients were positioned in a supine posture, and then a sphygmomanometer was placed on the forearm region. Briefly, patients were asked to rest for 20 min in supine position prior to data collection. Measurements were acquired using an ultrasound device (M‐Turbo, Sonosite, Seattle, WA, USA) performed in a longitudinal plane, positioned approximately 1–3 cm proximal to the antecubital fossa, with the arm abducted at approximately 80° from the body and the forearm in a supinated position. The ultrasound transducer (11 MHz) was positioned to visualize the lumen‐intima interfaces anterior and posterior while measuring the diameter or central flow velocity (pulsed Doppler) (Thijssen et al., 2019). After the initial images were recorded, the blood pressure cuff on the forearm was inflated to 200 mmHg for 5 min. After this time, arterial diameter was measured immediately following blood pressure cuff release for 3 min. The analyses were performed with the Brachial Analyzer software (Medical Imaging Applications LLC, Iowa, USA).

Absolute FMD (mm) ([diameter before cuff blood vessel − diameter after cuff]), percentage (%) FMD ([peak diameter − baseline diameter]/baseline diameter × 100), shear stress (SS) [(blood flow velocity (cm/s^−1^) × 8)/blood vessel diameter (mm)], FMD normalized [(FMD mm baseline) + (FMD %)/(SS)], and blood flow velocity (BFV) at baseline and immediately after cuff down were obtained. Endothelial dysfunction was defined as FMD less than or equal to 8% of the post‐cuff FMD (Thijssen et al., 2019).

### 2.8 Cardiopulmonary exercise testing

All tests were performed according to the American College of Cardiology and American Heart Association Guidelines (Fletcher et al., 2013), supervised by a physician and two previously trained physical therapists. All exercise tests were performed on a cycle ergometer with electromagnetic braking (Corival Recumbent, Medical Graphics Corp., USA). Metabolic gasses were evaluated using the Oxycon Mobile respiratory gas analyzer (Mijnhardt/Jäger) which was properly calibrated for volume and gasses for each test.

The protocol consisted of the following: (1) 5‐min rest period while sitting on the cycle ergometer; (2) 1‐min exercise at free‐wheel and 60 rotations per minute (rpm); (3) incremental phase with an increase of 5–20 Watts/minute until exhaustion; (4) 1‐min active recovery at free‐wheel; and (5) 5‐min passive recovery resting in sitting position. Twelve‐lead electrocardiography (ECG) was continuously monitored throughout the test (WinCardio, Micromed, Brazil). The test was finished when subjects were pedaling at their maximum possible effort level (physical exhaustion) and reported at least 2 of the following criteria: (1) age‐predicted maximal heart rate (HR) (220‐[age]); (2) general/leg fatigue or dyspnea; (3) angina or electrocardiographic evidence of ischemia or malignant arrhythmia (ventricular tachyarrhythmia, ventricular fibrillation, bigeminism); or (4) the inability to maintain a pedaling rate of 60 rpm for 30s (Fletcher et al., 2013). The load prescription (W) was based on the recommendation of the American College of Sports Medicine, where load (W) = [(height − age) × 12] − [(150 + 6 × weight)]/100, and according to the reported exercise tolerance (Committee Members et al., 2002).

### 2.9 Ventilatory and hemodynamic measurements during CPET

During CPET, the following parameters were measured using the average of the values from the last 30 seconds of the exercise test: workload (W) (watts), peak oxygen uptake (V̇O_2_) (mL·kg^−1^·min^−1^), V̇O_2_ (mL/min), V̇O_2_ (%predicted), carbon dioxide output (V̇CO_2_) (mL/min), RER: respiratory exchange ratio, V̇E (l/min), tidal volume (L), respiratory ratio (rpm), respiratory rate/tidal volume, the V̇E/V̇CO_2 slope_ was obtained to determine the relation between minute ventilation and carbon dioxide output, HR peak (bpm), OUES: linear relation between oxygen uptake and minute ventilation (Baba et al., 1996), and peak and rest of systolic and diastolic blood pressures (SBP and DBP, mmHg). Circulatory power (CP) was calculated as the product of peak V̇O_2_ and peak SBP (Cohen‐Solal et al., 2002), and ventilatory power (VP) was calculated by dividing peak systolic blood pressure by the V̇E/V̇CO_2slope_ (Forman et al., 2012). O_2_ pulse was calculated using the product of peak V̇O_2_ and peak HR. V̇O_2_/WR was determined by the relationship between maximal workload obtained and V̇O_2_ peak. In addition, V̇O_2_/W_slope_ was calculated through the relationship between V̇O_2_ and W in the test. Arterial oxygen saturation was measured non‐invasively by pulse oximetry (SpO_2_, %). The Borg scale for perceived exertion from 0 to 10, known as CR10 Scale (Category Ratio 10), was applied to quantify the perception of effort for lower limbs and for dyspnea (Borg, 1982).

### 2.10 Statistical analysis

Descriptively, results were presented as mean ± standard deviation, median and interquartile range, and percentage values, as appropriate. Initially, the Shapiro–Wilk test was performed to assess data normality, and Levene's test was used to assess homogeneity. When the data did not exhibit homogeneity of variances, Welch test correction was applied in the main analyses. For group characteristic analyses, the *X* ^2^ test and ANOVA one‐way test post hoc Tukey test were utilized. ANCOVA tests were employed to analyze CPET variables and FMD variables, adjusted for BMI, SBP, and HR due to the fact that these variables showed differences between the groups in baseline characteristics and are potential confounding factors for other analyses, followed by post hoc Tukey testing. Pearson's correlation test was employed to assess correlations between vascular reactivity e FMD (%), with CPET variables. A reference of *r* values as 0.1–0.3, 0.4–0.6, and 0.7–0.9 indicating small, moderate, and strong correlations, respectively. Furthermore, in the same outcomes, simple linear regression test was employed to assess the relationship between the two variables in the regression, and the *r* ^2^ was used. For non‐normal analysis, Kruskal–Wallis was used. An effect size (Cohen's F) was used to assess CPET and vascular reactivity analyses, in which 0.1–0.24 indicated a small effect size, 0.25–0.39 indicated a moderate effect size, and >0.4 indicated a large effect size. A *p*‐value <0.05 was considered statistically significant for all tests. SPSS software version 23.0 (IBM, Chicago, USA) and G*Power software version 3.1 (University of Dusseldorf, Dusseldorf, Germany) were used for calculations, and GraphPad Prism software version 8.0 (GraphPad, California, USA) was used for creating the images.

## 3 RESULTS

**FIGURE 1:**
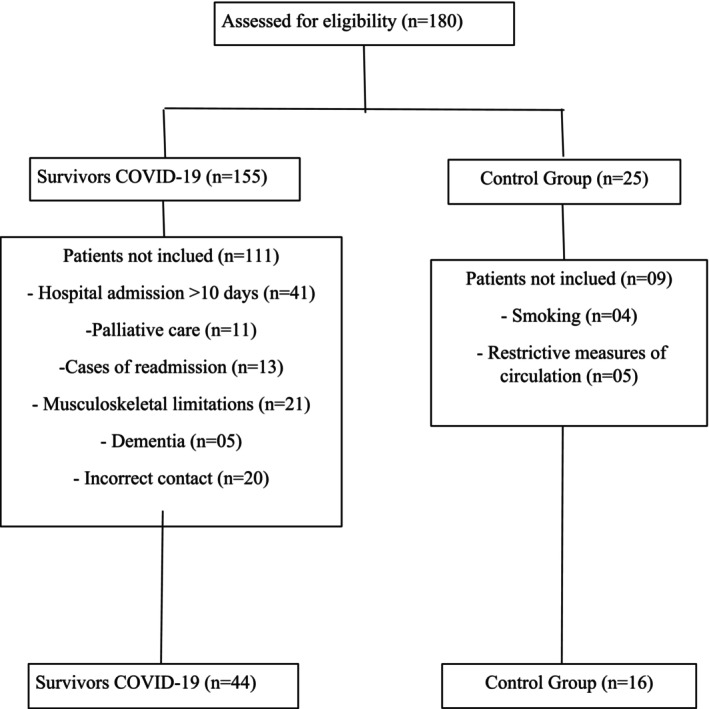
Flowchart.

One hundred and eighty individuals were initially recruited; after stratification, an age‐ and sex‐matched cohort was established before allocating them into groups based on COVID‐19 severity.: survivors of COVID‐19 with mild, moderate, and severe cases (*n* = 155), and a control group consisting of 25 participants (see Figure 1). Survivors of COVID‐19 were excluded for the following reasons: (1) forty‐one patients with hospitalization exceeding 10 days; (2) eleven patients on palliative treatment; (3) thirteen patients were readmitted to the hospital for other illnesses; (4) twenty‐one patients had musculoskeletal limitations; (5) five cases of dementia post‐hospital discharge; and (6) 20 patients were excluded for not responding to phone calls. In the control group, due to the pandemic situation in Brazil, five volunteers did not undergo assessments due to restrictive measures, and four smoking volunteers were excluded at the time of evaluation. In total, 44 survivors of COVID‐19 were evaluated. Additionally, 16 healthy volunteers matched for sex and age were allocated to the control group.

Table 1 presents sample characterization data for the groups of COVID‐19 survivors and the CG. All patients were matched for age and sex. Increased BMI values were observed for mild and moderate cases subgroups compared to the control group. Regarding lung function, it was not possible to observe obstructive or restrictive patterns. However, moderate cases and severe cases groups presented with reduced FVC and FEV_1_% values compared to the mild cases and control group. Regarding symptomatology, the main symptoms reported were fatigue, dyspnea, myalgia, hyposmia, dysgeusia, and headache. Patients who survived COVID‐19 had more comorbidities, mainly hypertension and diabetes, compared to the control group. We did not observe significant differences in the number of medications between survivors and the control group.

**TABLE 1:** Clinical characteristics and pulmonary function of COVID‐19 survivors and control group.

	Control (*n* = 16)	Mild (*n* = 16)	Moderate (*n* = 14)	Severe (*n* = 14)	*p*‐Value
Age (years)	49 ± 9	47 ± 9	49 ± 9	50 ± 6	0.85
Sex (male)	7 (9)	9 (7)	7 (7)	8 (6)	0.86
BMI (kg.m^2^)	24 ± 3	31 ± 5^c^	27 ± 4	30 ± 6^c^	**<0.001**
Race (Black)	13 (3)	15 (1)	13 (1)	10 (4)	0.28
Hospitalization^a^					
>1 week	–	–	64%	15%	**0.04**
<1 week	–	–	36%	85%	
Pulmonary Function					
FVC (%)	97 ± 10	104 ± 14	93 ± 16	85 ± 15^d^	**0.014**
FEV_1_ (%)	103 ± 10	109 ± 11	89 ± 18^c^, ^d^	85 ± 14^c^, ^d^	**<0.001**
FVC/FEV_1_ (%)	0.82 ± 0.4	0.79 ± 0.5	0.82 ± 0.5	0.81 ± 0.7	0.22
Persistent Symptoms					
Fatigue	–	9%	9%	12%	0.68
Dyspnoea	–	2%	7%	10%^c^, ^d^	**<0.001**
Myalgia	–	4%	10%	10%^c^, ^d^	**<0.001**
Hyposmia	–	7%	9%	8%	0.76
Dysgeusia	–	9%	5%	9%	0.87
Headache	–	5%	6%	8%	0.72
Medications					
Anti‐hypertensive	–	5%	–	4%	0.34
Beta‐blockers	–	20%	12%	16%	0.12
Antidepressant		6%	21%	14%	0.22
Anti‐hyperglycemic	–	13%	29%	32%	0.87
Comorbid^b^					
Asthma	–	6%	7%	7%	0.76
Hypertension	–	19%	36%	50%	0.95
Depression	–	6%	21%	14%	0.22
Diabetes	–	13%	29%	35%	0.72

*Note*: ANOVA One‐way test for continuous variables and chi‐squared test for categorical variables.

Abbreviations: BMI, body mass index; kg/m^2^, kilogram per square meter; FEV_1_, forced expiratory volume in 1 s; FVC, forced vital capacity; L/s, liters per second.

^a^ Analysis only between Moderate and Severe.

^b^ Analysis only between Mild, Moderate, and Severe.

^c^ Difference with control group.

^d^ Difference with the mild cases.

**FIGURE 2:**
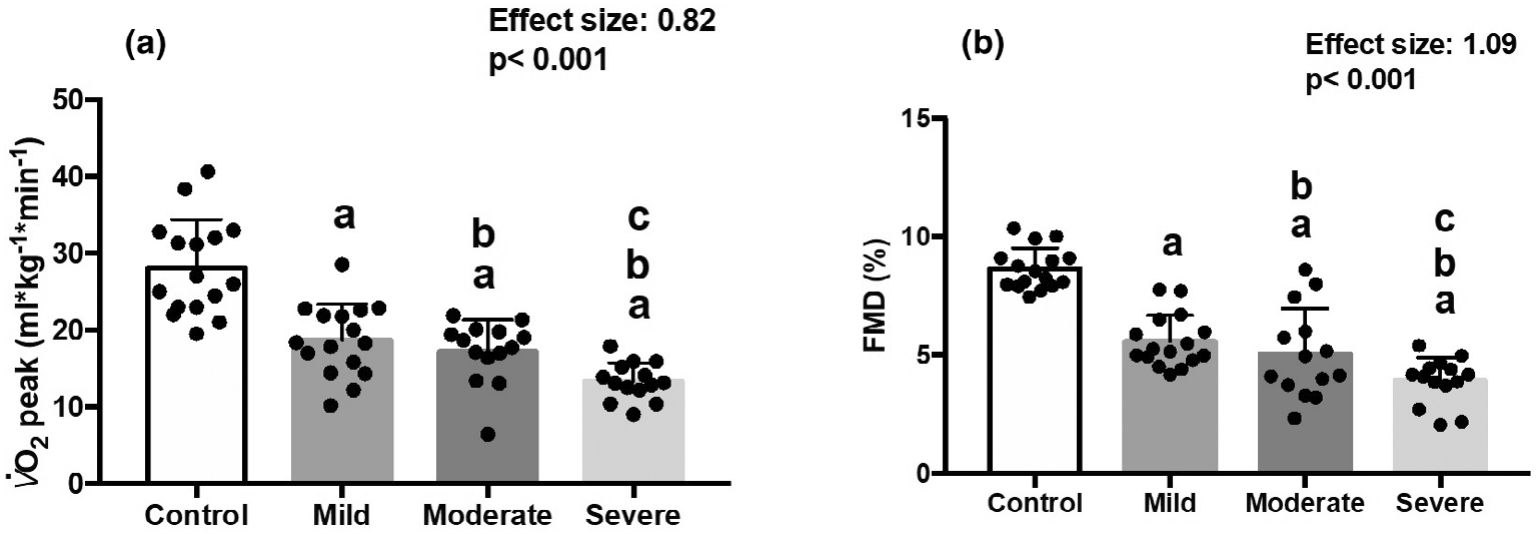
V̇O_2peak_ (mL/kg/min) and FMD in COVID‐19 survivors and CG. Used ANCOVA test for continuous variables. V̇O_2_, Oxygen uptake; FMD, Flow‐mediated dilation. ^a^Difference with control group; ^b^Difference with mild cases; ^c^Difference with moderate cases. Cohen's F as effect size. *p*‐Value adjusted by BMI, SBP, and HR, Post hoc Tukey.

Figure 2 illustrates the significant effect for V̇O_2peak_ (mL·kg^−1^·min^−1^) (effect size 0.82‐Cohen's F) and FMD (%) (effect size 1.09‐Cohen's F as vascular endothelial reactivity, which were primary outcomes). Regarding V̇O_2peak_, a significant decline was observed in all COVID‐19 severity subgroups 1 month after infection compared to the control group. Furthermore, severe and moderate cases survivors exhibited worse V̇O_2peak_ compared to mild cases. Interestingly, we observed a similar pattern in FMD (%) between survivor groups, with significantly lower values compared to the control group. Furthermore, severe cases showed worse endothelial dysfunction when compared to moderate cases and mild cases. We observed no difference between mild and moderate cases.

In Table 2, a significant reduction in the values of V̇O_2peak_, V̇CO_2_, V̇E, and test time is evident in all disease severities 1 month after infection, when compared to the control group. Although SpO_2_ differences between groups were significant. We observed significant differences between severe and moderate cases compared to survivors of mild cases and the control group for the variables tidal volume, respiratory ratio, and respiratory ratio/tidal volume (*p* < 0.01). Although we did not observe differences in the V̇E/V̇CO_2slope_, the severity of the disease appears to influence ventilatory performance.

**TABLE 2:** Symptoms limited exercise test variables. Mean ± Standard deviation.

	Control (*n* = 16)	Mild (*n* = 16)	Moderate (*n* = 14)	Severe (*n* = 14)	Adjusted *p*‐value*	Effect size^a^
Variables						
Load (Watts)	167 ± 62	128 ± 38^b^	104 ± 24^b^	106 ± 32^b^	**0.015** *	**0.46**
Time (s)	668 ± 52	643 ± 78	548 ± 45^b^, ^c^	600 ± 74^b^	**<0.001** *	**0.78**
Metabolic responses						
V̇O_2_ (mL.min)	2005 ± 774	1512 ± 569	1381 ± 292^b^	1422 ± 418^b^	**0.03** *	**0.40**
V̇CO_2_ (mL.kg)	2237 ± 810	1599 ± 709^b^	1548 ± 412^b^	1456 ± 440^b^	**<0.001** *	**0.60**
RER	1.14 ± 0.04	1.08 ± 0.15	1.07 ± 0.07	1.02 ± 0.10^b^	**0.01** *	**0.88**
V̇O_2_/W	12 ± 1.8	12 ± 4.2	13 ± 2.0	14 ± 4.7	0.28	0.25
Ventilatory responses						
V̇_E_ (l.min)	67 ± 21	50 ± 15^b^	44 ± 8^b^	45 ± 12^b^	**0.01** *	**0.48**
V̇_E_/*V̇*CO_2slope_	32 ± 2.8	30 ± 7.8	35 ± 5.5	34 ± 4.7	0.058	0.34
VP	6.10 ± 0.7	7.0 ± 2.0	5.9 ± 1.3	6.3 ± 0.9	0.134	0.27
OUES	2.9 ± 1.2	2.8 ± 0.6	2.2 ± 1.1	2.5 ± 0.9	0.058	0.37
SpO_2peak_	97 ± 1.1	95 ± 2.8	95 ± 1.7^b^	94 ± 1.7^b^	**<0.001** *	**0.62**
Hemodynamic responses						
HR_rest_ (bpm)	73 ± 14	79 ± 10	80 ± 14	90 ± 13^b^	**0.02** *	**0.64**
HR_peak_ (bpm)	165 ± 12	147 ± 35	144 ± 25	146 ± 26	0.058	0.31
SBP_rest_ (mmHg)	117 ± 7	130 ± 11^b^	126 ± 12	133 ± 10^b^	**<0.001** *	**0.69**
SBP_peak_ (mmHg)	196 ± 23	204 ± 27	202 ± 18	215 ± 21	0.058	0.17
DBP_rest_ (mmHg)	80 ± 5	81 ± 8	82 ± 8	85 ± 8	**<0.001** *	**0.75**
DBP_peak_ (mmHg)	90 ± 9.0	89 ± 13	92 ± 13	96 ± 12	0.43	0.17
V̇O_2_/W slope	10.0 ± 0.9	9.4 ± 0.9	8.2 ± 1.2^b^, ^c^	7.6 ± 1.5^b^, ^c^	**<0.001** *	**0.78**
O_2_ Pulse (mL/bpm)	12.2 ± 4.6	11.5 ± 3.3	9.9 ± 3.3	9.8 ± 2.7	0.43	0.02
CP (mmHg·mL^−1^·kg^−1^·min^−1^)	5598 ± 1751	3797 ± 971^b^	3537 ± 974^b^	2892 ± 692^b^	**<0.001** *	**0.57**
Perception of symptoms						
Borg lower body	7 [6–10]	6 [0–10]	7.5 [2–10]	8.5 [5–10]	0.20	0.07
Borg fatigue	4.5 [0–8]	5 [3–9]	6 [2–9]	6 [4–10]	0.09	0.10

*Note*: ANCOVA test for continuous variables and chi‐squared test for categorical variables. Post hoc Tukey.

Abbreviations: CP, circulatory power; DBP, diastolic blood pressure; ECG, electrocardiogram; HR, heart rate; OUES, linear relation between oxygen uptake and minute ventilation; PV, ventilatory power; RER, respiratory exchange ratio; SBP, systolic blood pressure; SpO_2_, peripheral saturation of oxygen; V̇CO_2_, carbon dioxide output; V̇E/V̇CO_2 slope_, linear relation between minute ventilation and carbon dioxide output; V̇E, ventilation; V̇O2, oxygen uptake; W, watts.

* *p*‐value adjusted by BMI, SBP, and HR.

^a^ Cohen's F.

^b^ Difference with the control group.

^c^ Difference with mild cases.

Regarding hemodynamic behavior variables, all surviving groups showed a lower CP compared to the control group. Furthermore, a significant reduction in V̇O_2_/W_slope_ was observed in moderate and severe cases compared to mild cases and CG, along with increased resting SBP values.

Regarding vascular reactivity in the different severities of COVID‐19 (Table 3), low FMD values (mm) were evident compared to the control group. Low absolute and normalized FMD values were also reported in this study. These variables reinforce the arterial change in the assessed conducting artery. Low SS in the moderate and severe case groups were observed in relation to the control group. Furthermore, we observed a negative impact on normalized FMD in mild cases and severe cases compared to the control group. Even so, moderate and severe cases survivors had lower SS values compared to the control group. No significant differences were observed in basal diameter between the groups.

**TABLE 3:** Vascular and endothelial reactivity. Mean ± Standard deviation.

	Control (*n* = 16)	Mild (*n* = 16)	Moderate (*n* = 14)	Severe (*n* = 14)	Adjusted *p*‐value*	Effect size^a^
Basal Diameter (mm)	3.9 ± 0.8	4.0 ± 0.8	3.7 ± 0.7	4.2 ± 0.9	0.38	0.20
FMD (mm)	0.44 ± 0.15	0.19 ± 0.07^b^	0.17 ± 0.06^b^	0.2 ± 0.10^b^	**<0.001** *	**0.99**
FMD normalized	0.006 ± 0.002^c^	0.002 ± 0.001	0.004 ± 0.002	0.007 ± 0.005^c^	**0.012** *	**0.46**
Shear stress	74.6 ± 17.8	72.5 ± 18.0	45.6 ± 12.2^c^	38.6 ± 19.2^b^, ^c^	**<0.001** *	**0.98**

*Note*: ANCOVA test for continuous variables was used. Post hoc Tukey.

Abbreviations: FMD, Flow‐mediated dilation.

* *p*‐value adjusted by BMI, SBP, and HR.

^a^ Cohen's F.

^b^ Difference with the control group.

^c^ Difference with mild cases.

**FIGURE 3:**
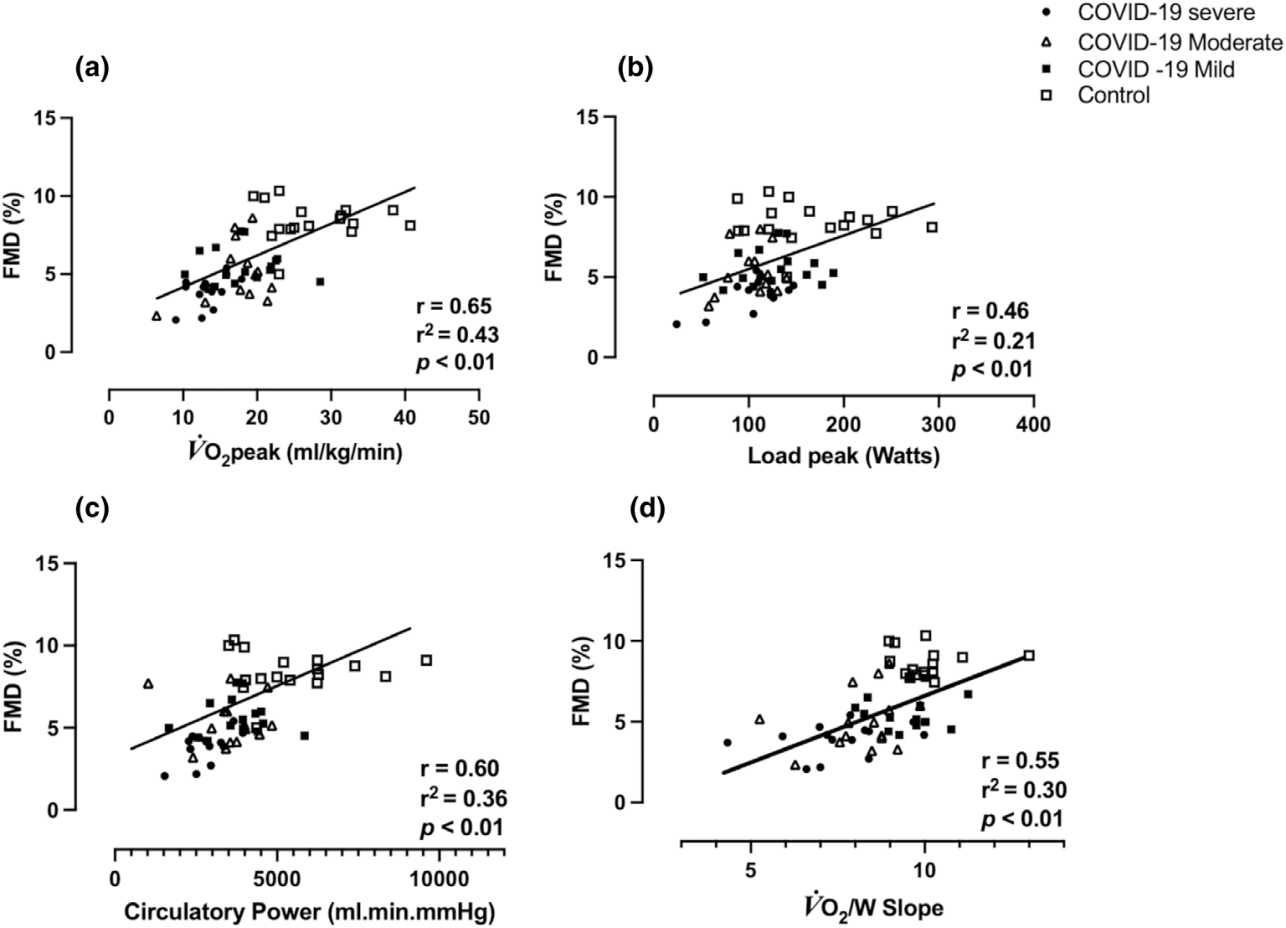
Correlations and Simple Regression between FMD% and variables analyzed during CPET variables Pearson's correlation test and simple regression test were employed to assess correlations between vascular reactivity as the primary outcome FMD (%), with cardiopulmonary test variables. FMD, Flow‐mediated dilation; V̇O_2_, Oxygen uptake.

Reductions in V̇O_2peak_, maximal load values achieved, CP, V̇O_2_/W_slope_ were all related to lower FMD (%) values (*r* = 0.65 strong, *r* = 0.46 moderate, *r* = 0.60 moderate, *r* = 0.55 moderate correlations, respectively). Additionally, FMD (%) correlated with 43% of the variance in V̇O_2peak_, a 21% reduction in W peak, 36% in CP, and 30% in vascular function during the test, as represented by the V̇O_2_/W_slope_ ratio (Figure 3).

## 4 DISCUSSION

To our knowledge, this is the first study to assess endothelial function and its relationship with CPET variables in survivors of mild, moderate, and severe COVID‐19 compared to healthy subjects. The main findings of this study were: (1) patients who survived COVID‐19 had a higher BMI, more comorbidities and medication use, and experienced more persistent symptoms 1 month after infection. (2) V̇O_2peak_ and FMD (%) respond similarly with worse values associated with the severity of COVID‐19; (3) all COVID‐19 survivors exhibited reduced exercise capacity, achieved lower exercise load levels, and finished exercise earlier. Additionally, COVID‐19 survivors showed a worse vascular response (V̇O_2_/W_slope_) after CPET when compared to the controls; (4) we observed worse endothelial function independent of COVID‐19 severity compared to the control group; and (5) worse FMD (%) was associated with lower V̇O_2peak_ and peak exercise workload values.

These findings have significant implications for the clinical management of COVID‐19 survivors. We observed a high incidence of persistent symptoms in our patients, with a particular focus on myalgia and dyspnea. Associations between disease severity were also observed for conditions such as hypertension, obesity, and diabetes. Early identification of at‐risk patients, such as those with high BMI and comorbidities, may enable the implementation of preventive interventions to reduce long‐term complications (Nasserie et al., 2021). Other studies have identified a similar pattern in these patients. Furthermore, promoting cardiovascular health and cardiopulmonary rehabilitation may be essential strategies in the post‐COVID‐19 recovery process (Ferreira et al., 2022; Subramanian et al., 2022).

We emphasize that all patients were stratified by disease severity, gender, and age, thus controlling for potential confounding factors. The results of this study highlight the clinical complexity of consequences 1 month after COVID‐19. Adults with higher BMI and comorbidities may be at greater risk of developing persistent symptoms and vascular complications after recovering from the infection (Chudzik et al., 2022). Additionally, the relationship between impaired endothelial function at rest and reduced exercise capacity suggests that cardiovascular health may be a key component in post‐COVID‐19 recovery (Ambrosino et al., 2022).

Endothelial cells have recently been implicated as the primary source of the onset and dissemination of acute respiratory distress syndrome (ARDS) caused by SARS‐CoV‐2, resulting in severe endothelial injury and widespread thrombosis (Gladka & Maack, 2020). It has been proposed that the loss of ACE2 is related to lung injury, where negative regulation and the decline of this enzyme may lead to dysfunction of the renin‐angiotensin system and potentially impair vascular function in COVID‐19 individuals (Gladka & Maack, 2020). The prothrombotic phenotype and diffused intravascular coagulation observed in COVID‐19 reflect endothelial dysfunction, which stimulates thrombosis, leading to exposure of prothrombotic subendothelial material, platelet aggregation, regulation of coagulation cascades, activation of thrombin and fibrin production, as well as alterations in vascular smooth muscle tone and blood flow (Roberts et al., 2020).

Endothelial dysfunction at the microcirculatory level may contribute to cardiovascular, pulmonary, and autonomic dysfunction in COVID‐19 patients, leading to changes in energy metabolism and organ perfusion. On the other hand, it is important to note that while acute physical inactivity can increase basal shear rate in large arteries, prolonged bed rest may decrease shear rate in the microcirculation (Pons et al., 2020). However, we highlight that in our study, patients did not remain in bed for extended periods, and they were matched for hospitalization or quarantine days. It is worth noting that hospitalized subjects received daily respiratory and ambulatory physiotherapy.

Therefore, the relationship between endothelial function and CRF could be explained by the endothelium's ability to regulate blood flow to muscle groups during exercise (Ambrosino et al., 2022). Endothelial dysfunction can limit oxygen delivery to active muscles during exercise, resulting in early fatigue and reduced performance. For the first time, we have shown that low FMD% values correlated with 43% of peak V̇O_2_ behavior, 21% of peak WR reduction, 36% of CP, and 30% of vascular function during the test, as measured by V̇O_2_/WR _slope_, varying from mild to severe COVID‐19. This demonstrates that changes in the systemic and pulmonary vascular endothelium could be related to the reduced peripheral vascular behavior during CPET, which can be considered an important factor in limiting physical exercise (Figure 3).

Recently, Ambrosino et al. (2022) highlighted that in survivors of severe to critical COVID‐19 cases, limitations in functional capacity may persist after 2 months of the initial infection, and these limitations may not depend solely on physical deconditioning but also on lower ventilation‐perfusion efficiency in these patients. The authors noted that low FMD values were associated with worse CPET performance and lower exercise ventilatory efficiency, as evidenced by high V̇E/V̇CO_2 slope_ and lower P_ET_CO_2_ values (Ambrosino et al., 2022). Additionally, in a study published by our research group, we observed a similar pattern of exercise limitation in survivors of mild to moderate cases. We found that reduced carbon monoxide diffusing capacity (DLCO) values were associated with worse peak V̇O_2_, P_ET_CO_2 peak_, O_2_ pulse peak, load values, and V̇E, V̇E/V̇CO_2 slope_, indicating impaired ventilatory function during exercise in COVID‐19 survivors (Back et al., 2022). In this context, our results indicate that lower FMD (%) values are strongly and moderately correlated with reductions in key performance metrics such as peak V̇O_2_, maximal load, CP, and V̇O_2_/WR _slope_, emphasizing the significant impact of vascular function on exercise capacity and performance variability.

When considering the relationship between endothelial function and exercise capacity in COVID‐19 patients, recent studies, such as those by Osburn et al. (2022) and Wang et al. (2020), highlight that endothelial function may be compromised in these individuals, particularly among survivors of severe cases. It has been speculated that even survivors of mild cases of COVID‐19 experience impairments in endothelial function and aerobic capacity (Durstenfeld et al., 2022). Endothelial dysfunction in COVID‐19 patients may be associated with a range of cardiovascular complications. Therefore, it emphasizes the importance of enhancing aerobic capacity to improve vascular function in these patients. This connection between aerobic capacity and vascular function may have significant implications for cardiovascular health management, especially in individuals facing conditions like COVID‐19. In summary, scientific literature suggests a positive relationship between endothelial function and exercise capacity, highlighting the benefits of regular exercise in enhancing vascular health. Applying these findings to specific populations, such as COVID‐19 patients, can provide valuable insights for management and rehabilitation strategies.

In this study, we have incorporated findings from the literature, showing an association between FMD and vascular behavior during exercise. Consequently, our preliminary findings deserve attention as they pertain to adults who have survived COVID‐19 in all degrees of severity. Thus, this association between endothelial dysfunction and CPET limitations in the post‐acute phase may indicate a greater focus on cardiovascular health for COVID‐19 survivors.

## 5 LIMITATIONS

Our study presented inherent limitations due to its cross‐sectional nature, such as having been conducted at a single city center, and we did not evaluate patients' baseline in relation to medications and endothelial function. However, the procedures adopted for COVID‐19 treatment and the rehabilitation protocol were similar across the institutions where the study took place, ensuring reliability regarding the impact of hospital rehabilitation on the functional capacity of our patients. Secondly, our data cannot be extrapolated to the general population of COVID‐19‐affected patients, as a significant portion of severe cases involve older individuals known to have more musculoskeletal dysfunctions and compromised immunity. In this study, older adults were excluded, and therefore, cases that resulted in longer hospitalizations and had a significant impact on functional capacity were also excluded. Our findings are limited to the adults 60 years or younger with mild to severe cases of the disease and hospital stays of less than 10 days, thus eliminating the bias of hospitalization duration on functionality impact. We observed a high prevalence of comorbidities that could influence our results, particularly cardiovascular comorbidities in FMD. However, we emphasize that this is a common characteristic among COVID‐19 survivors, regardless of the disease's severity. Finally, we did not conduct a functional assessment at the time of hospital discharge, but all volunteers were monitored during their hospitalization and contacted by phone for health condition follow‐up on the seventh and fourteenth day after the illness.

## 6 CONCLUSION

In conclusion, we highlight that endothelial function is compromised in COVID‐19 patients regardless of its severity. Endothelial dysfunction is closely associated with key variables from cardiopulmonary testing, thereby underscoring the importance of enhancing CRF to improve vascular function. These findings may underscore the need to maintain or improve CRF in these patients through physical exercise programs and participation in early rehabilitation programs as a crucial strategy to reduce cardiovascular risks.

## CONFLICT OF INTEREST STATEMENT

The authors declare no conflicts of interest. This study was supported by Fundação de Amparo a Pesquisa do Estado de São Paulo FAPESP (#2015/2650‐1 and #2020/15726‐0). Fundação de Amparo a Pesquisa do Estado do Amazonas PCTI EMERG_SAUDE II (#006/2020). CAPES‐PROEX (#001). CNPq (303885/2021‐1) and, CNPq/MCTI (#10/2023). This study was approved by the Institutional Medical Ethics Committee protocol #32408720.5.0000.5504.

## Data Availability

After publication, all data will be uploaded to the Institutional Repository of the Federal University of São Carlos and will be available at this link: https://repositorio.ufscar.br/.
